# A cross-sectional survey of mental health clinicians’ knowledge, attitudes, and practice relating to tobacco dependence among young people with mental disorders

**DOI:** 10.1186/s12913-014-0618-x

**Published:** 2014-11-26

**Authors:** Meghana Kulkarni, Lisa Huddlestone, Anne Taylor, Kapil Sayal, Elena Ratschen

**Affiliations:** Division of Epidemiology & Public Health, The University of Nottingham, Clinical Sciences Building, City Hospital Campus, Nottingham, NG5 1 PB UK; Nottinghamshire Healthcare NHS Trust, Nottingham, UK; Division of Developmental Psychiatry, The University of Nottingham, Queens Medical Centre, Nottingham, UK

**Keywords:** Smoking, Mental illness, Tobacco, Addiction, Mental health services

## Abstract

**Background:**

Mental health services in England are smoke-free by law and expected to provide comprehensive support to patients who smoke. Although clinicians’ knowledge in this area is reported to be limited, research exploring the issue in Child and Adolescent Mental Health Services (CAMHS) is lacking. This study aimed to investigate the knowledge, attitudes, and practice of clinicians working within specialist and highly specialist Child and Adolescent Mental Health Services (CAMHS) relating to tobacco dependence, its treatment and its relation to mental disorder.

**Methods:**

A cross-sectional survey of clinicians working across all CAMHS teams of a large UK National Health Service mental health Trust.

**Results:**

Sixty clinicians (50% response rate) completed the survey. Less than half (48.3%) believed that addressing smoking was part of their responsibility, and half (50%) asserted confidence in supporting patients in a cessation attempt. Misconceptions relating to smoking were present across all staff groups: e.g. only 40% of respondents were aware of potential interactions between smoking and antipsychotic medications, although psychiatrists were more knowledgeable than non-medical clinicians (91.6% vs 27.1%; OR 3.4, p < .001). Self-reported attendance at smoking-related training was significantly associated with more proactive clinical practice.

**Conclusions:**

There is a need to improve clinicians’ knowledge, capacity and confidence in effectively identifying, motivating, supporting and treating young smokers in the context of treatment for mental disorders.

**Electronic supplementary material:**

The online version of this article (doi:10.1186/s12913-014-0618-x) contains supplementary material, which is available to authorized users.

## Background

The links between smoking and mental disorder are complex and strong, with smoking prevalence, tobacco dependence, and smoking-related disease and mortality being disproportionately high among people with mental disorders compared to the general population [1,2] causing and exacerbating existing health inequalities in this vulnerable group [3]. The strength of the association between tobacco dependence and mental disorders is determined by a multitude of factors that include neurobiological, genetic and, importantly, psychosocial and cultural phenomena. Recognising these links gains particular importance in the context of smoke-free policies that were introduced in English National Health Service (NHS) mental health Trusts, which provide specialist treatment and care for people with mental disorders. Smoking cessation or abstinence, for example in the context of admission to an inpatient unit, can impact on drug metabolism and require monitoring to prevent potential drug toxicity [4]. While research to date has focussed on exploring and addressing smoking in adults with mental disorders, attention has recently been drawn to the importance of this topic in child and adolescent mental health services, both from a treatment and a prevention perspective [3].

There is evidence to highlight a strong association between mental disorders and smoking in adolescence [5,6], with young people with a diagnosis of emotional disorder, ADHD, or conduct disorder being more likely to smoke than their peers without these disorders [7,8]. In addition, the prevalence of smoking among individuals with first episode psychosis is six times higher than in matched aged and gender controls [5]. With most smoking behaviour initiated prior to adulthood [6], an increased likelihood of becoming heavier and more persistent smokers, and the contribution of tobacco dependence to increased health inequality [2,9], the Royal College of Physicians and the Royal College of Psychiatrists in the UK recommend targeted approaches for children and young people with emotional and behavioural disorders and highlight an urgent need for the identification of young smokers and the co-ordinated assessment and treatment of tobacco dependence in the context of treatment for emerging mental disorders [3].

Recently, the UK government committed to strategies aiming to improve physical health outcomes among people with mental disorders [10]. In this context, the introduction of smoke- free policies within mental health trusts has been described as an avenue towards promoting a smoke- free lifestyle [11]. Recent guidance from the National Institute for Health and Clinical Excellence [12] stipulates the provision of comprehensive, evidence-based treatment and support for patients with mental illness who smoke. Despite this and evidence that smokers with mental illness are similarly as motivated to quit as smokers without mental illness, and can successfully do so when supported appropriately [13], challenges in addressing smoking in mental health settings have been highlighted. Research within adult mental health services revealed that clinical staff are integral to the implementation of smoke-free practices, yet staff attitudes were found to be ambivalent [14-16]. Evidence suggests that that clinicians based in adult mental health inpatient settings may not be sufficiently equipped to support patients in addressing their smoking behaviour effectively, often reporting a lack of awareness and knowledge of tobacco dependence, its treatment, and interaction with mental disorders [15,17,18], including the fact that quitting smoking has been shown to improve symptoms of mental illness (rather than exacerbate them, as commonly assumed) [19].

To our knowledge, no research has been carried out to explore issues relating to smoking and treating smoking in the context of child and adolescent mental health services specifically. Responding to calls to address this knowledge gap, this study investigates mental health professionals’ knowledge and attitudes relating to smoking, its treatment, and its links with mental disorder, as well as clinical practice of addressing tobacco dependence among children and young people accessing Child and Adolescent Mental Health Services (CAMHS) in the UK.

## Methods

### Settings and participants

All clinicians working within a specialist CAMHS in one of the UK’s largest National Health Service (NHS) mental health trusts were invited to participate the paper based survey. In England, treatment and care for children and adolescents (up to 18 years of age) who have significant mental health problems or disorders is organised through a tier system, according to severity of symptoms and conditions from Tier 1 (primary care) to Tiers 3 and 4 (specialist and highly specialist (mainly inpatient) support). The teams included in the survey comprised several tier 3 “Looked After Children” i.e. children under the care of social care services; Neurodevelopmental Psychiatry; Paediatric Liaison; Substance Misuse and Early Intervention Psychosis; Specialist Community CAMHS; and Self-harm) and tier 4 (Adolescent In-patient and Intensive Intervention) teams. The names of all clinical staff involved in patient care were obtained from team leaders and personalised invitations including questionnaires posted to all staff. Survey completion was not incentivised, however responses were encouraged by advertising in staff areas, email cascade from the service managers, and in team meetings. A follow-up letter and questionnaire were sent to all non-respondents after four weeks. Ethical approval for the study was obtained from The University of Nottingham School of Medicine Ethics Committee and permission to conduct the research was given by Nottinghamshire Healthcare NHS Trust.

### Study instrument

Based on the instrument used in a previous study [15], a questionnaire was designed to explore mental health professionals’ knowledge, attitudes, and clinical practice related to tobacco dependence, its treatment and links with mental disorders Additional file 1. Twenty seven items relating to clinician characteristics, including training participation; clinician knowledge; the interplay between tobacco smoke and mental disorder; carcinogenic properties of tobacco smoke; symptoms of nicotine withdrawal; and clinician attitudes and practice were assessed by closed and multiple choice questions, as well as Likert scales. Clinician knowledge was assessed by asking respondents to give estimates of smoking prevalence within both the general and mental health populations and clinicians were asked to indicate their response of “true”, “false” or don’t know” to questions relating to the carcinogenic properties of tobacco smoke and the interplay between tobacco dependence and mental disorders. In the assessment of respondents’ knowledge of the symptoms of nicotine withdrawal, participants were asked to select the correct symptoms from a list of twenty possible symptoms. Options were based upon the Diagnostic and Statistical Manual of Mental Disorders, fourth edition (DSM-IV) diagnostic criteria for nicotine withdrawal (dysphoric or depressed mood; insomnia; irritability; frustration or anger; anxiety; difficulty in concentrating; restlessness; and decreased heart rate) and these were supplemented by seven additional symptoms of nicotine withdrawal (decreased tremor, cold symptoms, mouth ulcers, decreased caffeine metabolism, constipation, light headedness and weight gain) that have been identified in smoking research [20]. Five false options (e.g. headache) were included. Clinicians’ attitudes and practices in terms of addressing smoking with their patients were assessed by asking respondents to rate their agreement with a series of statements on a 5-point Likert scale (strongly agree to strongly disagree); respondents were then asked to rate on a 10-point numerical scale how important they felt it was to address patients’ smoking in the context of treatment for mental disorders, and how confident they felt in supporting patients to effectively quit smoking (scales ranged from 1 = not at all important/confident to 10 = extremely important/confident).

### Procedures

Survey responses were coded, entered, and analysed using IBM SPSS (version 21). Staff groups were categorised for analysis into medical (psychiatric consultants and junior doctors) and non-medical staff (nurses, psychologists, systemic therapists, other therapists and other health professionals). Where significant differences were detected between these groups, medical and non-medical staff groups were sub-divided for more detailed analysis (doctors, nurses, psychologists, and other therapists). Other a priori categories for analysis were based on reported smoking status (smokers vs. non-smokers/ex-smokers), age (under 49 years and over 49 years, as per median age), gender, whether respondents worked in a community setting or if they were ward or hospital based (adolescent in-patient team and paediatric liaison team), and reported training attendance relating to smoking in the past two years. The responses to the 5-point Likert scale items were dichotomised for analysis, with strong agreement and agreement forming one category and undecided, disagreement and strong disagreement forming the other. To assess levels of participants’ knowledge in relation to tobacco dependence, its treatment and its relation with mental illness, responses true, false, and “don’t know” were coded into two categories: correct and incorrect categories, with the answer “don’t know” being coded as incorrect. Performance relating to knowledge of the symptoms of nicotine withdrawal were ranked to obtain an overall measure of performance by aggregating points as follows: +1 (correctly identified symptoms), 0 (correct symptoms not identified) and −1 (incorrectly identified symptoms). The overall percentage of correct answers was also calculated.

### Data analysis

Descriptive analysis was performed to obtain means, medians, standard deviations, and proportions. Univariate analysis of categorical and continuous data was performed using Chi-squared tests (Fishers Exact Test where appropriate) and analyses of variance (ANOVA), or in the case of non-normal distribution of data, Mann–Whitney U-tests, respectively, to detect differences (taken to be significant at *p* ≤0.05) in outcomes between the subgroups. No statistically significant differences were detected for more than one sub-group for any outcome measure and, as such, multivariable analysis was not performed. Unless stated explicitly, significant differences between groups were not detected for individual outcome measures. All statistically significant results from univariate analysis are reported with odds ratios (OR), and 95% confidence intervals for sub-groups.

### Ethical approval

Favourable ethical opinion was obtained from the University Of Nottingham School Of Medicine Research Ethics Committee (L06062013 CHS EPH) and NHS research governance approval was granted by the Trust.

## Results

### Participant characteristics

The questionnaire was mailed to all 120 clinicians working in the specialist CAMHS, and after one follow-up mailshot, the response rate was 50% (n = 60). The survey was returned by 48 (47.5% response rate) non-medical staff and 12 (63.2%) medical staff. Of the 97 clinicians working in community based roles, 52 (53.6%) responded to the survey; of the 23 clinicians in ward based roles, 8 (34.8%) completed surveys were received. The predominance of female respondents (66.7%) reflects the fact that female clinicians comprised 77.5% (n = 93) of the overall sample. The median age range of respondents was 45–49 years. and four respondents (6.7%), all nurses, identified themselves as current smokers. Less than half (41.7%) of all respondents reported completing a Trust provided e-learning training module in relation to smoking. Differences in responses were not associated with gender, age range, or smoking status. Further demographic information relating to study participants can be found in Table 1.

**Table 1:** **Characteristics of survey respondents**

**Demographic details**			**Number of respondents (%)**
Professional group	Non-medical staff	Nurses	20 (33.3)
		Occupational therapists	2 (3.3)
		Psychologists	6 (10.0)
		Systemic therapists	5 (8.3)
		Other therapist	9 (15.0)
		Other health professional	6 (10.0)
	Medical staff	Consultant	9 (15.0)
		Junior doctor	3 (5.0)
		Adolescent in-patient	5 (8.3)
		T3 Community CAMHS	26 (52.0)
		Self-harm team	3 (5.0)
		Paediatric Liaison team	3 (5.0)
		Early intervention psychosis and substance misuse team	5 (8.3)
		Looked after children	12 (20.0)
		Neurodevelopmental team	2 (3.3)
Smoking status		Intensive Interventions team	4 (6.7)
		Current smoker	4 (6.7)
		Ex-smoker	17 (28.3)
		Never smoker	39 (65.7)
Training attendance	Training		25 (41.7)
	No Training		35 (58.3)

### Clinician knowledge

Overall, respondents believed that 30% (SD 17.1) of the general UK adult population, and 70% (SD 12.1) of the population of adults with mental disorders smoked and over three-quarters (79.6%) of participants knew that Nicotine Replacement Therapy (NRT) could be used as an aid for patients who wanted to reduce their tobacco consumption, but who did not want to quit.

### The interplay between tobacco smoke and mental disorder

Less than half (40%; n = 24) of respondents were aware that patients who smoke heavily can require higher doses of psychotropic medication, whereas medical staff were significantly more aware of this fact than their non-medically trained colleagues [91.6% vs 27.1% (OR = 3.4; 95% CI 2.1-5.5); p < .001]. Again, less than half (40.%) of all respondents were aware that serum levels of certain psychotropic medications can rise if patients stop smoking, and medical staff were significantly more likely to be aware of this metabolic link than non-medical staff [83.3% vs 29.1% (OR = 2.4; 95% CI 2.1-2.6); p < .001]. Further details concerning clinicians’ awareness of tobacco dependence and its treatment in the context of mental disorders can be found in Table 2.

**Table 2:** **Correct responses assessing clinician knowledge of tobacco dependence, its treatment, and its relation to mental illness**

**Statement**	**All respondents**	**Medical staff [n = 12; (%)]**	**Non-medical staff [n = 48; (%)]**	**Sig [F, p]**
Nicotine Replacement Therapy can interfere with psychotropic medication (false)	23	6 (50.0)	17 (35.4)	*F* = 2.25, p = .139
Addiction to NRT is common (false)	26	7 (58.3)	19 (39.5)	*F* = .038, *p* = .846
Patients who smoke require higher doses of certain psychotropic medication (true)	24	11 (91.6)	13 (27.1)	*F* = 22.59, *p*<.001*
If patients stop smoking serum levels of psychotropic medication can rise (true)	24	10 (83.3)	14 (29.1)	*F* = 12.71, p = .001*
NRT can be used as an aid for smokers who want to reduce their tobacco consumption (true)	47	10 (83.3)	37 (77.1)	*F* = .886, *p* = .351
The recording of patients smoking status is mandatory (true)	55	10 (83.3)	45 (93.7)	*F* = 1.57 , *p* = .217

*Denotes statistical significance at p < = 0.05.

### Carcinogenic properties

The great majority (88.3%) of respondents were aware that “tar” is a carcinogenic component of tobacco smoke, while over one third (38.3%) of clinicians believed that carbon monoxide causes cancer and one-fifth (20%) of respondents thought that nicotine is a carcinogen.

### Symptoms of nicotine withdrawal

In relation to respondents’ awareness of the symptoms of nicotine addiction, the median number of correctly identified symptoms was 7 from a possible 15 correct answers (46.6%). Both incorrect and correct answers were aggregated as described in methods, and a mean score of 5.8 (SD 2.3) out of a maximum of 15 was achieved overall.

### Clinician attitudes and practice

Sixty percent of respondents felt that addressing tobacco dependence among young people with mental disorders was important; staff provided an overall mean rating of 6.27 (SD 2.1) and less than half (48.3%) of the respondents felt that addressing patients smoking was within their remit of responsibility as a mental health professional. The vast majority of staff (86.7%; n = 52) did not feel that addressing patients’ smoking would have an adverse impact on the therapeutic relationship, or that quitting smoking during treatment for their mental disorder would have a negative impact on their recovery (81.4%; n = 48). Over half (53.3%) of all respondents felt that smoking was an important coping mechanism for patients, helping them to deal with their mental disorder and those clinicians who reported attending smoking cessation training were significantly more likely to agree [80.0% vs 34.3% (OR = 2.1; 95% CI 1.4-3.5); p = .001]. Half (50.0%) of all respondents reported confidence in their ability to support patients in a quit attempt effectively, providing a mean confidence rating of 5.6 (SD 2.4) out of a maximum of 10, with clinicians who had attended smoking cessation training scoring themselves significantly higher than those who had not (6.4 vs. 5.1; p = .051).

Nearly two-thirds (62.7%) of respondents reported regularly assessing patients’ smoking status within their working practice; the non-medical group were more likely to do so in comparison to the medical group. Over one-third (36.8%) reported routinely asking patients about their motivation to quit or reduce their smoking, whereas those clinicians who reported attending training were significantly more likely to do so than those who had not [54.1% vs 24.2%; (OR = 2.3; 95% CI 1.1-5.1); p = .028]. Less than one third (29.8%) of respondents reported signposting or referring patients to the local “stop smoking” service and medical staff were less likely to refer or signpost patients, in contrast to their non-medical colleagues. Again, smoking cessation training attendance was associated with the practice of referring or signposting motivated patients to the local stop smoking service compared to non-attendance [50.0% vs 15.1%; (OR = 1.4, 95% CI 1.1-1.8); p = .008]. Less than one-quarter (22.4%) of respondents reported nicotine replacement therapy and behavioural support to be readily available in their clinical setting, whereas those respondents working in ward environments were significantly more likely to report the availability of cessation support than clinicians based in the community [62.5% vs 16.0%; (OR = 3.9; 95% CI 1.7-8.9); p = .010]. Nearly all clinicians (91.6%) were aware that the recording of smoking status in electronic patient records was mandatory practice in the Trust. Table 3 provides further details of the association between training and respondents’ attitudes and clinical practice.

**Table 3:** **The influence of training on clinical practice and attitudes relating to addressing tobacco dependence among young people with mental illness**

**Agreement (strongly agree/agree) (%)**				
**Statement**	**All respondents**	**Training attended**	**Training not attended**	**Sig (2-sided)** **[** ***X*** ^***2***^ **,** ***p =*** **]**
I feel it lies within the remit of my responsibility as a mental health professional to address patients smoking	51.6	64.0	48.8	*X* ^*2*^ = 2.61, *p* = .088
I routinely assess patients smoking status	62.7	62.5	62.8	*X* ^*2*^ = .001, *p* = .595
I routinely ask patients about their motivation to quit smoking	36.8	54.1	24.2	*X* ^*2*^ = 5.34, *p* = .028*
I routinely signpost/refer patients to local stop smoking services	29.8	50.0	15.1	*X* ^*2*^ = 8.06, *p* = .008*
Access to stop smoking medication and support are readily available in my clinic/on my ward	22.4	16.6	26.4	*X* ^*2*^ = .778, *p* = .526
Smoking is an important coping mechanism for patients.	53.3	80.0	34.3	*X* ^*2*^ = 12.24, *p* = .001*
Patients stopping smoking while on my ward/in my clinic would not interfere with recovery	81.3	75.0	85.7	*X* ^*2*^ = 1.08, *p* = .328
Addressing patients smoking would not have an adverse effect on the therapeutic relationship	86.6	88.0	85.7	*X* ^*2*^ = .066, *p* = 1.00

*Denotes statistical significance at p=<0.05.

## Discussion

This study reveals gaps in overall knowledge with regard to tobacco dependence, its treatment, and its relation with mental disorder among clinicians working within specialist child and adolescent mental health services in England. Due to the overall adequate response rate to the survey of 50%, the inclusion of all clinical professions, the focus on both community and in-patient services, and the size of the Trust, the results are likely to be applicable to other CAMHS in England, However, despite this, a number of study limitations are noted. Generalizability may be limited by survey-typical selection bias and reporting bias, which may be reflected in the circumstance that self-reported smoking status among the respondents was noticeably lower (6.7%) than the national average (21%) of smoking prevalence among the UK adult population, and also lower than the rates described in similar studies of mental health professionals [15,20]. This, in combination with an overall small sample size, meant that certain subgroup analyses were only meaningful in parts and may be one reason why, in contrast to previous research among adult mental health clinicians [15,17,18], our results failed to highlight many differences between professional groups, and did not allow for the exploration of differences according to smoking status. Yet, to the best of our knowledge, this is the first study that explores smoking related knowledge and practice in child and adolescent mental health specialist settings and thus makes an important contribution to the literature.

Whilst a greater proportion of staff than previously shown in a sample of adult mental health clinicians [15] agreed that addressing tobacco dependence was important, the rating of importance was moderate. The majority of respondents felt that it was not within their remit of responsibility as a mental health professional, indicating that staff attitudes remain overall ambivalent as shown previously [15,17,18]. A perceived divide in responsibilities between physical and mental health care, despite moves towards a more holistic approach to patient care [14], appears to persist. Levels of confidence in effectively supporting patient in a quit attempt were generally found to be low. However, attending training appeared to be associated with improved practice in terms of asking patients about their motivation to quit, and signposting or referring them for specialist smoking cessation treatment.

Like previous research [15,20] among adult mental health clinicians illustrating prevailing misattributions regarding smoking and mental illness (for example the perception of the importance of tobacco for self-medication), this study found that over half of respondents considered smoking an important coping mechanism for patients in dealing with their mental disorder. This suggests that there is a lack of awareness relating to the importance of smoking cessation for people with mental disorder [19], which may contribute to reluctance among staff to address tobacco dependence with their patients. However, despite this, the great majority of staff did not believe that patients stopping smoking while being treated for a mental disorder would have an adverse effect on the therapeutic relationship or that it would interfere with recovery, which should be viewed as positive signals in the light of the historic smoking culture in mental health settings [4,16].

Accurate knowledge is essential if clinicians are to provide patients with appropriate advice and support in relation to smoking and smoking cessation. In this survey, gaps in knowledge in relation to smoking, smoking cessation, and nicotine withdrawal were apparent across all staff groups, indicating that clinician capacity to address tobacco dependence among young people is likely to be limited. Importantly, a lack of knowledge and awareness in relation to the metabolic interactions between components of tobacco smoke and antipsychotic medication is of particular importance from a clinical perspective, in case patients quit (or take up) smoking [3]. While the majority of psychiatrists were informed of the issue (which demonstrates a notable improvement to the earlier survey conducted in adult mental health settings [15] other clinical groups were not, indicating a need for training across all groups. This consideration is supported by the circumstance that reported training attendance, despite being low at around 50%, was associated with more accurate knowledge and more frequent signposting and referrals to specialist smoking cessation support. CAMHS nurses and other non-medical clinicians, particularly those working in the community, are likely to have a greater number of contacts with young patients and are thus perhaps more likely to identify, advice, and provide support for young smokers than psychiatrists. A lack of awareness and erroneous beliefs concerning smoking and smoking cessation may reduce their motivation to address tobacco dependence within this population, thus missing important opportunities for health promotion.

If tobacco dependence among young people with mental disorders is to be addressed, then the effective identification and support of young smokers is essential. Commissioning strategies within this and many English mental health Trusts have gone some way towards ensuring that young smokers accessing specialist CAMHS are identified. However, few clinicians appear to follow inquiries about smoking status with an assessment of the young person’s motivation to quit and their interest in receiving support, and fewer still reported routinely signposting or referring patients to local stop smoking services. This in combination with the lack of confidence reported by clinicians to provide support to smokers, means that opportunities for intervention are currently being missed. The accessibility of NRT and cessation support is influenced by current commissioning strategies. Within the Trust studied, and possibly in other Trusts in England, only in-patient services are incentivised to provide NRT and associated support, while community teams tend to refer or signpost patients to local smoking cessation services. This practice is exemplified by our finding relating to the reported accessibility of NRT in the respective clinical settings. The effects of this practice on young people accessing community teams may have consequences in terms of successful cessation attempts. A delay in providing patients with cessation support could result in a loss of motivation and represents a missed opportunity to improve physical and mental health and could undermine effort to improve health inequality.

## Conclusions

To the best of our knowledge, this is the first study to assess clinicians’ knowledge, practices, and attitudes relating to smoking, smoking cessation and its relation to child and adolescent mental health services. Our findings highlight the need to increase the capacity of mental health professionals to address tobacco dependence within the context of the treatment of mental disorders among young people. Opportunities for smoke-free promotion and smoking cessation are currently likely to be missed. Commissioning strategies should incentivise all child and adolescent mental health services to identify, advise and effectively support (including the prescribing of NRT) young smokers. For this to be successful, staff require comprehensive development to improve knowledge and attitudes, and support for clinicians is required to ensure that interventions to support smoking cessation within CAMHS do not negatively impact on existing clinical priorities. Further research is required to assess the feasibility and effectiveness of interventions targeted at addressing smoking among young people with significant mental disorders.

### Consent

Informed consent was obtained from the participants for the publication of this report.

## Additional file

Additional file 1:
**Clinician survey.** A copy of survey questionnaire distributed to clinicians.
