# Supplementary material for: A cross-sectional survey of mental health clinicians’ knowledge, attitudes, and practice relating to tobacco dependence among young people with mental disorders
Source: BMC Health Serv Res. 2014 Nov 26;14:618. doi: 10.1186/s12913-014-0618-x (PMC4251854; doi:10.1186/s12913-014-0618-x)
Supplement: Additional file 1: — Clinician survey. A copy of survey questionnaire distributed to clinicians. [file 12913_2014_618_MOESM1_ESM.doc]

| **Please indicate which specialist CAMHS services you work in (tick as many as appropriate).** | | | | | | | | | | | | | | | | |
| --- | --- | --- | --- | --- | --- | --- | --- | --- | --- | --- | --- | --- | --- | --- | --- | --- |
| Adolescent In-patient Unit | | |  | | Head 2 Head team | | | | | | | | |  | | |
| City & County South Tier 3 CAMHS Team | | |  | | Looked After Children Team | | | | | | | | |  | | |
| County North Tier 3 CAMHS Team | | |  | | Neuro-developmental Team (NDT) (including ADHD) | | | | | | | | |  | | |
| Self-harm Team | | |  | | Intensive Interventions Team | | | | | | | | |  | | |
| Paediatric Liaison | | |  | |  | | | | | | | | | | | |
| **Information about yourself** | | | | | | | | | | | | | | | | |
| **1** | Age Range : (tick as appropriate)  20-24 25-29 30-34 35-39 39-44 45-49 50-54 55-59 60-64 65-69 | | | | | | | | | | | | | | | |
| **2** | Gender | | Male  Female | | | |  | | | | | | | | | |
| **3** | Professional group: please choose from below | | | | | |  | | | | | | |  | | |
|  | Registered nurse | |  | | | | Consultant psychiatrist (substantive/ locum ((substantiative | | | | | | |  | | |
|  | Occupational therapist | |  | | | | Junior Doctor | | | | | | |  | | |
|  | Psychologist | |  | | | | Trainee psychologist | | | | | | |  | | |
|  | Systemic therapist | |  | | | | Other health professional | | | | | | |  | | |
|  | Other therapist (Art/ play) | | \|  \| \| --- \| | | | |  | | | | | | | | | |
|  |  | |  | | | |  | | | | | | |  | | |
| **4** | Smoking status | | | | | | Current smoker | | | | | | |  | | |
|  |  |  |  |  |  |  | Ex-smoker (*go to Q 7*) | | | | | | |  | | |
|  |  |  |  |  |  |  | Never-smoker (*go to Q 7*) | | | | | | |  | | |
| **Smoking and smoking cessation** | | | | | | | | | | | | | | | | |
| **5** | What percentage of adults do you think are current smokers in the UK? | | | | | | | | | | | | | % | | |
| **6** | What percentage of adults with severe mental illness (such as schizophrenia) are current smokers in the UK? | | | | | | | | | | | | | % | | |
| **7** | Which of the following are symptoms of nicotine withdrawal? Please tick as many as appropriate. | | | | | | | | | | | | | | | |
|  | Restlessness | | |  | | Weight gain | | | | | | |  | | | |
|  | Anxiety | | |  | | Increased tremor | | | | | | |  | | | |
|  | Dysphoric or depressed mood | | |  | | Decreased tremor | | | | | | |  | | | |
|  | Decreased heart rate | | |  | | Increased appetite | | | | | | |  | | | |
|  | Insomnia | | |  | | Colds | | | | | | |  | | | |
|  | Sleepiness | | |  | | Mouth ulcers | | | | | | |  | | | |
|  | Difficulty concentrating | | |  | | Decreased caffeine metabolism | | | | | | |  | | | |
|  | Decreased appetite | | |  | | Headache | | | | | | |  | | | |
|  | Irritability, frustration or anger | | |  | | Diarrhoea | | | | | | |  | | | |
|  | Light-headedness | | |  | | Constipation | | | | | | |  | | | |
| **8** | Which of the following components of tobacco smoke cause cancer? Please tick as many as appropriate. | | | | | | | | | | | | | | | |
|  | Nicotine | Carbon monoxide | | | | Other smoke constituents known as ‘Tar’ | | | | | | | | | | |
| **9** | Please indicate your level of agreement with the following statements, on a scale from 1 to 5. **1 – strongly agree/ 2 – agree/ 3 – undecided/ 4 – disagree/ 5 – strongly disagree** | | | | | | | | | | | | | | | |
| **9a** | I feel it lies within the remit of my responsibilities to address patients’ smoking.  1 2  3  4  5 | | | | | | | | | | | | | | | |
| **9b** | I routinely assess patients’ smoking status within my working practice.  1 2  3  4  5 | | | | | | | | | | | | | | | |
| **9c** | I routinely ask patients about their motivation to quit/reduce their smoking with my working practices.  1 2  3  4  5 | | | | | | | | | | | | | | | |
| **9d** | I routinely signpost/refer patients to the local stop smoking services.  1  2  3  4  5 | | | | | | | | | | | | | | | |
| **9e** | On my ward/in my clinic, access to stop smoking medication and behavioural support are readily available for patients who want to stop or reduce smoking.  1  2  3  4  5 | | | | | | | | | | | | | | | |
| **9f** | Smoking is an important coping mechanism for many patients, helping them to deal with their mental health problems.  1 2  3  4  5 | | | | | | | | | | | | | | | |
| **9g** | Patients stopping smoking while treated on my ward/in my clinic would interfere with their recovery.  1  2  3  4  5 | | | | | | | | | | | | | | | |
| **9h** | Addressing smoking with my patients would have an adverse effect on the therapeutic relationship.  1  2  3  4  5 | | | | | | | | | | | | | | | |
| **10** | Please indicate whether you think the following statements are true or false. | | | | | | | | | | | | | | | |
|  |  | | | | | | | | | **True** | | **False** | | | | **Don’t know** |
| **10a** | Nicotine replacement therapies can interfere with psychotropic medications. | | | | | | | | |  | |  | | | |  |
| **10b** | Addiction to nicotine replacement products is common. | | | | | | | | |  | |  | | | |  |
| **10c** | Patients who smoke heavily need higher doses of certain psychotropic medications. | | | | | | | | |  | |  | | | |  |
| **10d** | If patients stop smoking, blood levels of psychotropic medications can rise. | | | | | | | | |  | |  | | | |  |
| **10e** | Some nicotine replacement products can be used as an aid for smokers who want to reduce their cigarette consumption but don’t want to quit smoking. | | | | | | | | |  | |  | | | |  |
| **10f** | Recording patients’ smoking status is mandatory on Rio. | | | | | | | | |  | |  | | | |  |
| **11** | On a scale from 1 to 10, how important do you feel is to address patients’ smoking in the context of treatment for mental illness? **1 – ‘not at all important’/ 10 – ‘extremely important’** | | | | | | | | | | | | | | | |
|  | Please insert a number between 1 and 10. | | | | | | | |  | | | | | | | |
| **12** | On a scale from 1 to 10, how confident would you feel in supporting patients who want to give up smoking effectively? **1- ‘not at all confident’ to 10 – ‘extremely confident’** | | | | | | | | | | | | | | | |
|  | Please insert number between 1 and 10. | | | | | | |  | | | | | | | | |
| **13** | In the last two years, have you received any training through the Trust related to treating smoking? | | | | | | | E-learning | | | Other | | | | None | |
|  |  |  |  |  |  |  |  |  |  |  |  |  |  |  |  |  |
| **14** | Finally, would you be willing to help us to develop and test interventions to help people reduce the harmfulness of their smoking? | | | | | | | Yes | | | No | | | | Unsure | |
|  |  |  |  |  |  |  |  |  |  |  |  |  |  |  |  |  |
